# Increased dendritic inhibition of dentate gyrus granule cells in a mouse model of Down syndrome

**DOI:** 10.3389/fncel.2026.1714586

**Published:** 2026-02-26

**Authors:** Nicole Gutmann, Ute Häussler, Anabel Mersi, Marie Follo, Josef Bischofberger, Jan M. Schulz

**Affiliations:** 1 Translational Epilepsy Research, Department of Neurosurgery, Medical Center – University of Freiburg, Faculty of Medicine, Freiburg, Germany; 2 Faculty of Biology, University of Freiburg, Freiburg, Germany; 3 Department of Biomedicine, University of Basel, Basel, Switzerland; 4 BrainLinks-BrainTools, University of Freiburg, Freiburg, Germany; 5 Department of Medicine I, Lighthouse Core Facility, Medical Center – University of Freiburg, Faculty of Medicine, University of Freiburg, Freiburg, Germany; 6 Roche Pharma Research and Early Development, Neuroscience and Rare Diseases Discovery, Roche Innovation Center Basel, F. Hoffmann-La Roche Ltd., Basel, Switzerland

**Keywords:** dendrite, GABA, hippocampus, inhibition, interneuron, optogenetics, patch-clamp electrophysiology, trisomy 21

## Abstract

Down syndrome is the most common genetic neurodevelopmental disorder associated with mild-to-moderate intellectual disability. A disturbed excitation-inhibition balance is thought to be a major cause for the intellectual deficits in DS. In this study, we used patch-clamp electrophysiology, optogenetic stimulation and immunohistochemistry to investigate synaptic inhibition from specific interneuron subpopulations onto granule cells of the dentate gyrus in Ts65Dn mice. Optogenetically evoked inhibitory postsynaptic currents (IPSCs) from somatostatin (SOM) interneurons onto dendrites of granule cells in the outer molecular layer (ML) did not differ between euploid (Eu) and Ts65Dn mice, indicating normal distal dendritic inhibition in Ts65Dn mice. In addition, optogenetically evoked IPSCs from parvalbumin (PV) interneurons were significantly reduced, indicating reduced functional somatic inhibition in Ts65Dn mice. In contrast, activation of cholecystokinin (CCK) positive interneurons by targeted electrical microstimulation of the inner molecular layer (iML) resulted in IPSCs of increased amplitude (Eu: 372.5 ± 51.97 pA, *n* = 10; Ts65Dn: 619.9 ± 74.68 pA, *n* = 9). GABAergic synaptic terminals of CCK interneurons express cannabinoid receptor 1 (CB1). Quantitative analysis of synapses double-labeled for CB1 and the vesicular GABA transporter (VGAT) revealed a significantly increased number of putative CCK interneuron terminals in the iML of Ts65Dn mice (Eu: 0.048 ± 0.014 puncta/μm^3^, Ts65Dn: 0.34 ± 0.12 puncta/μm^3^). In contrast, the density of PV-VGAT double-positive synapses within the granule cell layer did not differ between the two genotypes. Taken together, our results indicate that proximal dendritic inhibition from CCK interneurons is increased in the dentate gyrus of Ts65Dn mice, while PV interneuron-mediated somatic inhibition appears to be unchanged or functionally diminished.

## Introduction

Down syndrome (DS) is the most common genetic disorder of intellectual disability (Desai, 1997; Antonarakis et al., 2020). In addition to the neurological system, DS affects the cardiovascular and musculoskeletal systems including common phenotypes of short stature, muscle hypotonia and congenital heart defects. Importantly, patients have a significantly higher risk of suffering from epilepsy and early-onset Alzheimer (Antonarakis et al., 2020). The underlying genetic mechanism is a triplication of the human chromosome 21 causing a perturbation in gene regulation and expression (Dierssen, 2012; Antonarakis et al., 2020). There are currently no approved pharmacological treatments to address cognitive deficits in DS patients. The most widely used model to investigate the pathophysiological mechanisms underlying impairments in DS is the Ts65Dn mouse in which a segmental trisomy for the distal murine chromosome 16 was produced by reciprocal translocation. This segment hosts genes, which are evolutionarily conserved from human chromosome 21, including the DS critical region (Reeves et al., 1995). Ts65Dn mice recapitulate most DS phenotypes. In particular, they show the typical neurological disturbances like deficits in short- and long-term memory, reduced neurogenesis and impaired performance on complex visual spatial learning tasks (Chakrabarti et al., 2007; Dierssen, 2012).

An imbalance of excitation and inhibition in the trisomic brain is thought to be the main cause for the deficits in declarative learning and memory (Kleschevnikov et al., 2004; Chakrabarti et al., 2010; Rueda et al., 2020). Long-term potentiation (LTP), thought to be the synaptic mechanisms underlying memory, is impaired in Ts65Dn mice (Malenka and Nicoll, 1999; Kleschevnikov et al., 2004). Calcium influx through NMDA receptors is necessary for most forms of LTP in the hippocampus (Malenka and Nicoll, 1999), and NMDA receptor activation has recently been shown to be impaired due to increased dendritic inhibition in the CA1 area (Schulz et al., 2019). Importantly, application of non-competitive GABA_A_ receptor blockers (Pentylenetetrazol or Picrotoxin) in non-epileptic concentrations rescued the behavior in novel object recognition tasks and the induction of LTP (Kleschevnikov et al., 2004; Fernandez et al., 2007). Together, these results suggested that aberrantly increased GABA_A_ receptor mediated inhibition leads to abnormalities in hippocampus-dependent learning and memory in DS.

The dentate gyrus (DG) of the hippocampus is essential for memory processing as it contributes to pattern separation (Leutgeb et al., 2007; McHugh et al., 2007; Myers and Scharfman, 2011; Madar et al., 2019). Different interneuron subpopulations control its activity and output along the trisynaptic path (Hu et al., 2014; Elgueta and Bartos, 2019). In the DG of Ts65Dn mice, Kleschevnikov and colleagues found an increased frequency of miniature IPSCs (mIPSCs) and strongly increased electrically evoked GABA_A_- and GABA_B_-mediated IPSCs (Kleschevnikov et al., 2012). Which specific interneurons mediated this increase in inhibition could not be determined using the available approaches. More recent studies using cell type or layer specific activation of GABAergic inputs have shown that enhanced dendritic inhibition likely mediated by somatostatin (SOM) expressing interneurons contributes to altered inhibition in the CA1 area and the prefrontal cortex (PFC) (Schulz et al., 2019; de Zorilla San Martin et al., 2020). Therefore, we used optogenetic and targeted electrical stimulation of specific inputs from GABAergic interneurons in combination with immunohistochemistry to investigate synaptic inhibition from specific interneuron subpopulations onto granule cells of the DG in Ts65Dn mice in the present study. The particular focus was on SOM-, cholecystokinin (CCK)-positive, and parvalbumin (PV-) interneurons mediating distal dendritic, proximal dendritic and perisomatic inhibition.

## Materials and methods

### Animals

Electrical stimulation experiments were performed on Ts65Dn (B6EiC3Sn a/A-Ts (17^16^) 65Dn/J) mice obtained from The Jackson Laboratory. Ts65Dn females were backcrossed with B6C3F1/OlaHsd males (F1 hybrid males from C57BL/6JOlaHsd ♀ × C3H/HeNHsd ♂ obtained from Harlan). For optogenetic experiments, we used triple crosses of B6EiC3Sn a/A-Ts(17^16^)65Dn/J ♀ × F1 ♂ (B6.129P2-Pvalbtm1 (cre) Arbr/J × Ai32 (RCL-ChR2(H134R)/EYFP)) and B6EiC3Sn a/A-Ts(17^16^)65Dn/J ♀ × F1 ♂ (SST tm2.1(cre) Zjh/J × Ai32 (RCL-ChR2 (H134R)/EYFP)), referred to as Eu or Ts65Dn, in the remainder of the text. We identified the supernumerary chromosome in 17^16^ of Ts65Dn mice in the offspring by standard polymerase chain reaction (PCR) procedures (Reinholdt et al., 2011). Eu and Ts65Dn mice were littermates. In this study experiments were performed with 6-week-old and fully mature (8-week-old) mice of both sexes. Mice were housed in groups of up to 5 animals in standard ventilated cages with a 12 h light/dark cycle and had *ad libitum* access to food and water. All experiments were approved by the Basel Cantonal Committee on Animal Experimentation according to federal and cantonal regulations.

### Perfusion and slice preparation for immunohistochemistry

Five Eu (3 male, 2 female) and four Ts65Dn (2 male, 2 female) fully mature mice were transcardially perfused under deep anesthesia with 0.9% saline followed by paraformaldehyde (PFA, 4% in 0.1 M phosphate buffered saline (PB), pH 7.4). The brains were post-fixed overnight in PFA at 4 °C. Afterwards they were cryoprotected in 30% sucrose and frozen in isopentane. Frontal sections with a thickness of 50 μm were cut on a cryostat and stored at 4 °C in 0.1 M PB for immunohistochemistry.

### Slice preparation for patch clamp recordings

Mice (41 male, 22 female) were placed for approximately 10 min in an oxygen-enriched environment to increase cell survival. We anesthetized the animals with isoflurane (4% in O_2_, Vapor, Draeger). Decapitation was performed in accordance with the national and institutional guidelines as soon as the righting reflex was absent. Slices were cut as previously described (Geiger et al., 2002; Bischofberger et al., 2006): The brain was carefully removed in ice-cold sucrose-based solution that contained (in mM): 87 NaCl, 25 NaHCO_3_, 2.5 KCl, 1.25 NaH_2_PO_4_, 75 sucrose, 0.5 CaCl_2_, 7 MgCl_2_ and 10 glucose (equilibrated with 95% O_2_/5% CO_2_; Osmolarity: 325–328 mOsm). Parahorizontal slices of 350 μm thickness were cut with a VT1200 vibratome (Leica Microsystems) and collected in the same sucrose-based solution. Finally, they were incubated at 35 °C for 30 min and stored at room temperature until the experiments were performed.

### Patch clamp recordings

We visually identified granule cells (GCs) by using infrared differential interference contrast video microscopy. The slices were continuously superfused with ACSF at room temperature, containing the following (in mM): 125 NaCl, 25 glucose, 25 NaHCO_3_, 2.5 KCl, 1.25 NaH_2_PO_4_, 2 CaCl_2_ and 1 MgCl_2_ (equilibrated with 95% O_2_/5% CO_2_; Osmolarity: 318–320 mOsm). Patch pipettes were pulled from borosilicate glass tubing (outer diameter: 2.0 mm; wall thickness: 0.5 mm; Hilgenberg) on a Flaming-Brown P-97 puller (Sutter Instruments).

GCs were voltage-clamped at −80 mV in the whole-cell configuration. The current signal was measured using a Multiclamp 700A amplifier (Molecular Devices), low-pass filtered with a cutoff frequency of 8 kHz and digitized at 20 kHz via a CED Power 1401 Interface (Cambridge Electronic Design). Patch pipettes had a resistance in a range of 2-4 MΩ. The internal solution depended on the type of experiment. In general, we used potassium-chloride-based (KCl) internal solutions for the recording of proximal synaptic inputs and cesium-chloride-based (CsCl) internal solutions for the recording of distal synaptic inputs from SOM interneurons. Specifically, we used the following KCl-based solution with addition of a potassium-gluconate for greater stability in the initial experiments employing electrical stimulation of GABAergic inputs in the iML of 6-week-old mice (in mM): 52 KGluc, 100 KCl, 10 HEPES, 0.1 EGTA, 2 MgCl_2_, 2 Na_2_ATP, 1 Phosphocreatine, 0.3 GTP, 5 QX314 (bromide or chloride) and 0.1% biocytin, adjusted to pH 7.28 with KOH. Subsequently, we employed KCl-based solutions for electrical stimulation (in mM): 120 KCl, 10 HEPES, 2 MgCl_2_, 20 BAPTA, 2 Na_2_ATP, 0.3 GTP, 5 QX314 (bromide or chloride); and optogenetic stimulation of PV interneurons (in mM): 140 KCl, 10 EGTA, 10 HEPES, 2 MgCl_2_, 2 Na_2_ATP, 0.3 GTP, 5 QX314 (bromide or chloride) and 0.1% biocytin. The pH was adjusted to 7.3 with KOH and the osmolarity was approximately 315 mOsm/L. For recording of distal dendritic inputs evoked by optogenetic stimulation of SOM-interneurons, pipettes were filled with a CsCl-based solution with addition of cesium-gluconate (CsGluc) containing the following in (mM): 40 CsGluc, 100 CsCl, 10 EGTA, 10 HEPES, 2 MgCl_2_, 2 Na_2_ATP, 2 TEA-Cl, 0.3 GTP and 5 QX314 (bromide or chloride) and 0.1% biocytin, adjusted to pH 7.3 with CsOH.

Criteria to include recordings in the analysis were the following: initial seal resistances were at least 1 GΩ; series resistance (R_s_) changed less than 30% during the recording and did not exceed 20 MΩ. An online analysis was performed to monitor the R_s_ and input resistance (R_in_) during the experiments, by applying a − 5 mV voltage step at the end of each sweep. Furthermore, we excluded experiments, if IPSCs were recorded more than 7 h after slice preparation. All experiments were performed at room temperature (20–22 °C). Data was collected using IGOR Pro 6.31 (WaveMetrics) and the CSF library support from Cambridge Electronic Design.

### Extracellular synaptic stimulation

To stimulate synaptic inputs, pipettes with a resistance of 3–6 MΩ were filled with a HEPES-buffered Na^2+^-rich solution. The stimulating pipette was placed in the inner molecular layer (iML), close to the granule cell layer (GCL). Recordings in the electrical stimulation experiments were performed in presence of 3 mM Kynurenic acid (Kyn) to block glutamatergic receptors at glutamatergic synapses, which are also recruited by electrical stimulation to ensure that the recorded IPSCs were GABAergic. Two electrical stimuli of 0.2 ms length were applied at low intensities (10–30 μA) with an inter-stimulus-interval (ISI) of 200 ms.

### Channelrhodopsin-2 (ChR2) mediated activation of GABAergic interneurons

A diode laser (DL-473, Rapp OptoElectronic GmbH) was used in this study. The laser was coupled to the epifluorescence port of the respective microscope (Zeiss Examiner, equipped with a 63x NA = 1.0 water immersion objective; Carl Zeiss Microscopy GmbH) via fiber optics and were controlled via TTL pulses. Whole field stimulation was performed using light with a wavelength of 473 nm. Two pulses were applied (1 ms duration each). Due to different decay time constants of the evoked IPSCs, the ISI was adjusted to trigger a second response after the first had almost returned back to baseline (stimulation of PV interneurons: ISI = 200 ms; stimulation of SOM interneurons: ISI = 500 ms). The field of illumination was targeted either to the GCL, when stimulating PV interneurons, or the border region of the ML to the stratum lacunosum moleculare to stimulate dendritic inputs mediated by SOM interneurons. Recordings of SOM inputs were done in presence of 3 mM Kyn to block glutamatergic receptors, because some Cre-driver lines may have unspecific expression in excitatory neurons (Müller-Komorowska et al., 2020).

### Drugs and reagents

Picrotoxin (PTX, Sigma Aldrich) was dissolved at 50 mM in ethanol, Kyn (Tocris) at 300 mM in 0.6 N NaOH and gabazine (Tocris) at 10 mM in Millipore water. Drug aliquots were stored in the freezer at −20 °C. The dilutions of these drugs in ASCF were prepared at the day of the experiment at the following concentrations: 100 μM PTX, 3 mM Kyn and 10 μM gabazine.

### Immunohistochemistry

Acute slices (350 μm thick) with biocytin-filled cells were fixed overnight in 4% PFA and underwent the following protocol. First, they were washed in 0.1 M PB three times (15 min each). Blocking was done for 1 h at room temperature with the following solution (per slice): 475 μL 0.3% Triton X-100, 25 μL normal donkey serum (NDS). Next, the slices were transferred in the solution of the primary antibodies for 72 h at 4 °C (per slice): 475 μL 0.3% Triton X-100, 25 μL NDS, chicken anti-green-fluorescent-protein (GFP, 1:1000, #ab13970, Abcam). After another four washing steps (15 min each) in 0.1 M PB slices were put in the secondary antibody solution (per slice): 475 μL 0.3% Triton X-100, 25 μL NDS, donkey anti-chicken Alexa 488 (1:500, #703–545-155, Jackson ImmunoResearch), Streptavidin Alexa Fluor 568 (1:1000, #s11226, MoBiTech) and DAPI (1:10000, Sigma Aldrich). We incubated slices overnight at 4 °C. The slices were washed in another three steps (10 min-2*15 min) in 0.1 M PB. Slices were placed on microscope slides, liquid was removed with filter paper and they were mounted directly with ProLong Gold Antifade (Thermo Fisher Scientific).

Another protocol was used for immunohistochemistry of 50 μm fixed cryostat slices. After washing in 0.1 M PB, slices were preincubated with 0.25% Triton X-100 and 10% normal horse serum (NHS) in 0.1 M PB for 30 min at room temperature. Followed by incubation with primary antibodies 3 h at room temperature and 4 °C overnight: guinea pig anti-vesicular GABA transporter (VGAT, 1:500, #131004, Synaptic Systems), rabbit anti-PV (1:1000, #PV-27, Swant) and rabbit anti-cannabinoid receptor type 1 (CB1, 1:500, #258003, Synaptic Systems). Slices were transferred into the secondary antibodies with the following dilutions: donkey anti-rabbit Alexa 488 (1:500, #A21206, Thermo Fisher Scientific), donkey anti-guinea pig Cy5 (1:200, #706175148, Jackson ImmunoResearch), and counterstained with DAPI (1:10000, Roche Diagnostics). After incubating 3 h at room temperature, slices were washed in 0.1 M PB, mounted and coverslipped with DAKO fluorescence mounting medium.

### Imaging and 3D-reconstruction

Images of 350 μm thick slices were taken with a Zeiss LSM 700 confocal microscope. Zeiss ZEN software was used for the processing.

Cryostat slices (50 μm thick) were imaged with a Zeiss LSM 880 confocal microscope with AiryScan using a 40x water immersion (1.2 NA, additional 2x zoom factor) or a 63x oil immersion objective (1.4 NA, additional 2x zoom factor). Images were taken with a z-stack step size of 0.14 μm and had approximately a total thickness of 7 μm. We measured chromatic aberration with microscopic beads and could not find a distortion.

We imaged three slices per animal and four regions within each slice. In total 12 images per animal were analyzed. 3D-reconstruction was done in Imaris (Version 9.9.2 Bitplane). First, a baseline subtraction was performed on the VGAT channel. Next, puncta were automatically reconstructed with the spot analysis tool of Imaris. A mask of VGAT^+^ puncta was created in either the CB1 or PV channel, to mark all double positive synapses. A second automatic detection of VGAT^+^ and CB1^+^ or PV^+^ puncta was again performed with the spot analysis tool. Density of VGAT^+^ and double positive VGAT^+^ and CB1^+^ or PV^+^ puncta was calculated by dividing the total number of puncta by the volume of the whole z-stack.

### Data analysis

The analysis of patch clamp data was performed with the software Stimfit (https://neurodroid. Github.io/stimfit) (Guzman et al., 2014) and scripts written in Python. On mean traces, the amplitude of the two responses to stimulation was identified by measuring the peak over 5 sample points and subtracting the baseline formed by the mean over 1,280 sample points or 64 ms, respectively.

Input resistance and R_s_ were determined directly after break-in and through-out the experiment, respectively, by applying a − 5 mV step pulse. The baseline for those measurements was calculated as the mean over 1,800 sample points or 90 ms before the start of the −5 mV voltage step. To evaluate R_in_, the mean was calculated over a defined 50 ms time window, starting 100 ms after the onset of the voltage pulse. The difference between the calculated mean value and the described baseline was computed and used to calculate R_in_. To evaluate the R_s_, the onset peak amplitude was determined and subtracted from the baseline. Another evaluated cellular property was the decay time constant *τ*_(break-in)_. It was measured for the onset peak amplitude, in response to a − 5 mV voltage step directly after break-in. The time constant τ_(break-in)_ was estimated by the weighted average of time constants from a biexponential fit starting at 95% of the amplitude and ending approximately 20 ms after the onset of the voltage step. The estimate of the cell capacitance (C_p_) was calculated with τ_(break-in)_ and R_s_ directly after break-in.

We analyzed the risetime and decay time constant τ for IPSCs in response to a double pulse stimulation. The risetime was calculated from 20 to 80% of the peak amplitude for the first IPSC. A biexponential fit as for τ_(break-in)_ was used to determine the time constant τ of the second IPSC starting at 95% of the amplitude and ending 250 ms after the stimulation. τ represents the weighted average of the fit.

### Inclusion criteria for electrophysiological recordings

Neurogenesis in the DG continues well into adulthood, but declines with age (Ben Abdallah et al., 2010). New-born and immature GCs show striking differences of cellular and synaptic electrophysiological properties including the cellular input resistance compared to mature GCs (see, e.g., Heigele et al., 2016). We used the following strategy to obtain a homogenous sample of GCs to enable robust statistical evaluation of genotype-specific effects on evoked GABAergic inputs: 1. GCs were recorded from the outer GCL adjacent to the ML where primarily mature GCs reside. 2. Based on recordings of outer GCs obtained from 8-week-old mice kept in standard housing, we used the 95-percentile of the cumulative contribution of R_in_ of 550 MΩ as the cut-off threshold for the inclusion of GCs in our analysis (Supplementary Figure S1). R_in_ measurements were neither affected by the intracellular solution nor by the genotype, allowing us a pooling of R_in_ from all experiments (Supplementary Figure S1). In this GC population with R_in_ ≤ 550 MΩ, GCs can be considered to be relatively mature. Table 1 shows the cell parameters R_in_, *τ*_(break-in)_ and C_p_ for all cells recorded in 6- and 8-week-old mice. There were no significant differences between Eu and Ts65Dn mice at a comparable level of cellular maturity (*p* ≥ 0.11, unpaired t-tests two-tailed).

**Table 1:** Cellular properties of GCs recorded from Eu and Ts65Dn mice in the DG.

	Eu		Ts65Dn	
	R_in_ ≤ 550 MΩ (*n* = 102)	R_in_ > 550 MΩ (*n* = 26)	R_in_ ≤ 550 MΩ (*n* = 89)	R_in_ > 550 MΩ (*n* = 23)
R_in_ [MΩ]	341.5 ± 0.01	818.6 ± 51.3	322.3 ± 12.7	874.9 ± 73.1
τ_(break-in)_ [ms]	1.51 ± 0.03	1.51 ± 0.06	1.50 ± 0.04	1.60 ± 0.09
C_p_ [pF]	119.7 ± 2.55	119.2 ± 3.84	126.3 ± 3.14	122.2 ± 5.71

Values are shown as mean ± SEM. Including experiments done in all conditions and both ages of mice recorded from in the whole study.

### Experimental design and statistical analysis

Statistical analysis was performed in Prism 9 (GraphPad). Unpaired two-tailed t-tests were used to detect significant effects between two groups. We used a mixed-effect analysis to test for differences in the input–output relation with repeated measures and two-way ANOVA for non-repeated measures (e.g., comparison between the two groups of ages) or for comparison between stimulations of different interneurons. As there were no genotype-specific differences in synaptic properties, data was pooled for comparison between targeted interneurons. For data pooled across genotypes, one-way ANOVA was used. Multiple comparisons were performed using a Šídák-Test, which minimizes the occurrence of type one errors by a weighting of the *p*-value. The significance level was set to *p* = 0.05 for all tests. All data is shown as mean ± SEM. The number (n) of observations reflects for electrophysiological experiments the number of cells recorded from and for immunohistochemical experiments the number of animals. For electrophysiological recordings, blinding of the experimenter to the genotype was not possible, because Ts65Dn mice have phenotypic characteristics, for example a smoother scalp, smaller brain and body sizes. Although a formal randomization of experiments was not performed, Eu control mice were littermates of Ts65Dn mice and were always used in an interleaved fashion. For the 3D-reconstruction, the analysis images were randomized and the experimenter was fully blinded.

## Results

### Increased proximal dendritic inhibition evoked by iML stimulation in Ts65Dn mice

**Figure 1:**
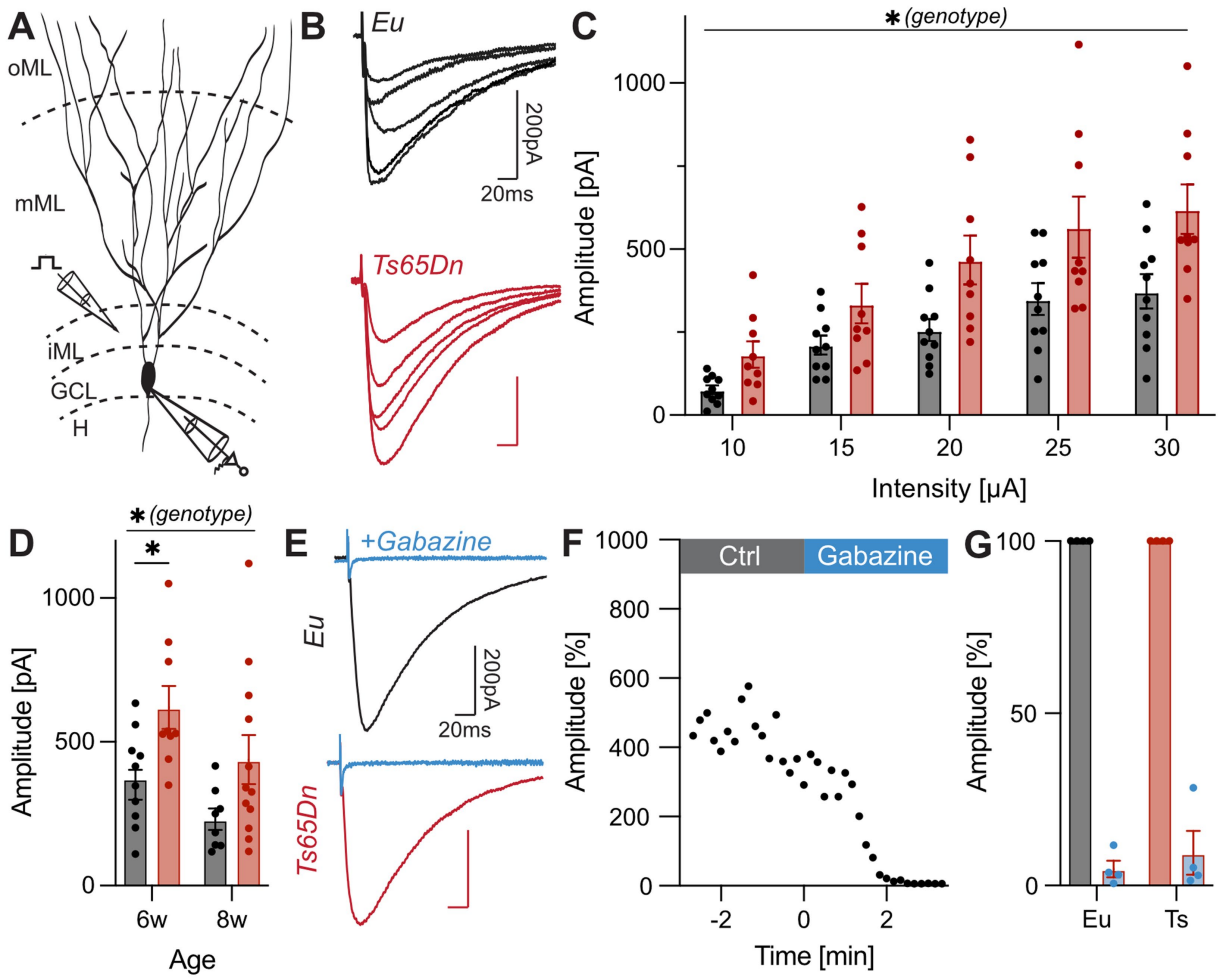
Electrical stimulation in the iML evokes IPSCs with an increased amplitude in Ts65Dn mice. **(A)** Schematic illustration of the stimulation in the iML and recording site in the GCL (modified from Lodge et al., 2020). **(B)** Representative mean IPSCs recorded in GCs of Eu and Ts65Dn 6-week-old mice. The holding potential was −80 mV. Recordings were performed in the presence of 3 mM Kyn. **(C)** Group data for the input–output relation. IPSC amplitude is plotted over stimulating current strength. Mixed-effect analysis (repeated measure) indicated a significant effect of the genotype (*p* = 0.0176, *F*_(1, 17)_ = 6.917, *n*_Eu_ = 10, *n*_Ts65Dn_ = 9). **(D)** Combined data of IPSCs from 6- and 8-week-old mice evoked with a stimulation intensity of 30 μA. Significant effect of the genotype reveals increased IPSC amplitude in Ts65Dn mice (*p* = 0.002, *F*_(1, 36)_ = 11.46, two-way ANOVA, *n*_Eu_ = 19, *n*_Ts65Dn_ = 21). **(E)** Representative traces for IPSCs evoked by injecting 20 μA. Wash-in of 10 μM gabazine for Eu and Ts65Dn mice completely blocked the response. **(F)** The IPSC amplitude is plotted over time. Time of the wash-in of gabazine is indicated. After approximately 2 min the response was suppressed. **(G)** Evaluation of the reduction of IPSC amplitudes in presence of gabazine for Eu (*n* = 4) and Ts65Dn mice (*n* = 4). A two-way ANOVA demonstrated a significant effect between control and presence of gabazine (*p* < 0.0001, *F*_(1, 12)_ = 751.30). The response was significantly reduced by up to 95% in the presence of gabazine (Eu = 95.2 ± 2.4%; Ts65Dn = 90.5 ± 6.3%; *p* < 0.0001, *Sidak’s multip*le-comparisons test). Ctrl, control; Eu, euploid; GC, granule cell; GCL, granule cell layer; H, hilus; iML, inner molecular layer; IPSC, inhibitory postsynaptic current; Kyn, kynurenic acid; mML, medial molecular layer; oML, outer molecular layer.

Previous studies demonstrated enhanced GABAergic synaptic transmission in the hippocampus of Ts65Dn mice (Kleschevnikov et al., 2012; Schulz et al., 2019). Kleschevnikov and colleagues showed increased inhibitory synaptic signaling in the DG of Ts65Dn mice when they stimulated electrically in the molecular layer (ML) while recording from GCs. The identity of the interneurons mediating the increased inhibition in Ts65Dn mice has not been determined. In order to stimulate specifically local inhibitory inputs onto the proximal dendrites of GCs, we used electrical stimulation at low intensity in the inner ML (iML) in two cohorts of mice of different ages (Figure 1; Supplementary Figure S2). These experiments as well as subsequent experiments employing optogenetic stimulation were performed under the assumption that there were no major changes in axonal excitability of targeted interneurons in the DG of Ts65Dn mice.

Inhibitory postsynaptic currents were evoked at increasing current intensities ranging from 10 μA to 30 μA. Amplitudes of evoked IPSCs increased with higher stimulation intensities in Eu and Ts65Dn mice (*p* < 0.0001, *F*_(2, 34)_ = 57.01, mixed-effect analysis, *n*_Eu_ = 10, *n*_Ts65Dn_ = 9) (Figure 1C). There was a significant effect of the genotype indicating larger IPSCs in Ts65Dn mice (*p* = 0.0176, *F*_(1, 17)_ = 6.917, mixed-effect analysis). To test for differences in fully mature mice, experiments were repeated in a cohort of 8-week-old mice. The results were qualitatively very similar (Supplementary Figure S2), with a strong trend toward an effect of the genotype by stimulation intensity interaction (*p* = 0.08, *F*_(1, 18)_ = 3.39, mixed-effect analysis; Supplementary Figure S2). Combining the IPSCs evoked at 30 μA from both datasets supported a significant effect of the genotype (*p* = 0.002, *F*_(1, 36)_ = 11.46, two-way ANOVA, n_Eu_ = 19, n_Ts65Dn_ = 21; Figure 1D). Experiments were performed in the presence of Kyn to block glutamatergic neurotransmission. Wash-in of the GABA_A_ receptor blocker gabazine after the experiment caused a full suppression of the electrically evoked IPSCs, confirming that the recorded currents were GABAergic (Figures 1E–G). Together, these results support the hypothesis of an increased inhibition in the DG of Ts65Dn mice and point to proximal dendritic inhibitory inputs as an important source.

### Age-dependent reduction of optogenetically activated SOM and PV interneuron inhibition in the dentate gyrus of Ts65Dn mice

**Figure 2:**
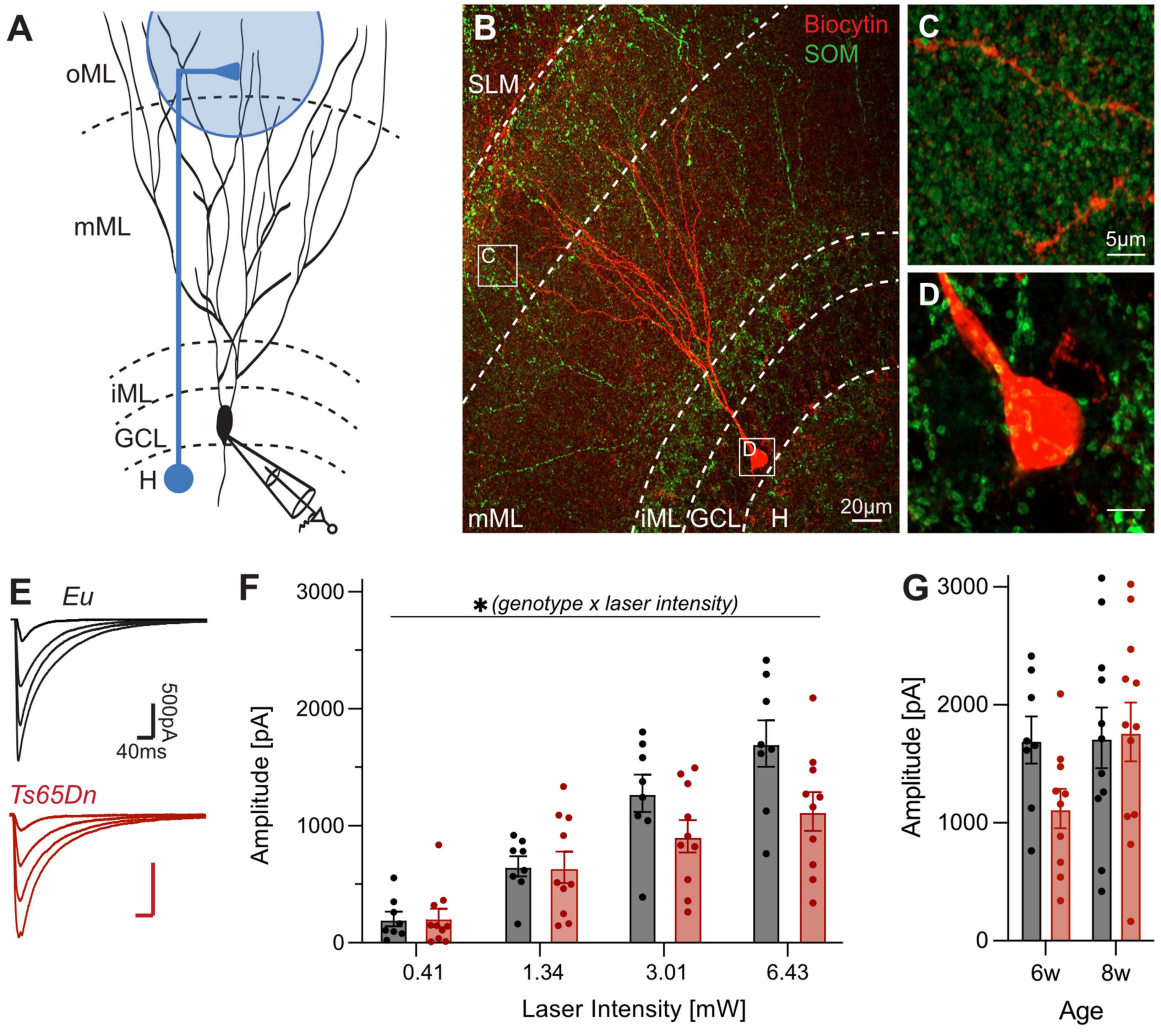
Optogenetically evoked SOM inputs onto the distal dendrites of GCs are not enhanced in Ts65Dn mice. **(A)** Experimental design. A patch-clamp recording was obtained from a GC. Optogenetic stimulation in the oML was used to activate synaptic inputs from SOM interneurons indicated by a blue schematic with its soma in the hilus and an axon targeting the distal dendrites of GCs in the oML. GCs were recorded at a holding potential of −80 mV in presence of 3 mM Kyn. **(B)** Biocytin-filled GC. Neuronal processes of SOM interneurons are shown by the YFP signal in green. **(C)** Putative SOM interneuron synapses enclose the pictured distal dendrite of the GC in the oML. **(D)** The negligible YFP signal in the GCL indicates that the soma of GCs is not the main synaptic target of SOM interneurons. **(E)** Mean traces of GC recordings after optical activation of SOM interneuron synapses in Eu and Ts65Dn 6-week-old animals. **(F)** Averaged data representing the input–output relation. The amplitude of IPSCs increases with increasing laser intensity. Mixed-effect analysis (repeated measure) revealed an effect of the laser intensity and genotype interaction (*p* = 0.0012, *F*_(3, 48)_ = 6.175, two-way ANOVA, *n*_Eu_ = 8, *n*_Ts65Dn_ = 10). **(G)** Data combined from recordings in two cohorts of 6- and 8-week-old mice in response to the highest laser stimulation intensity of 6.43 mW. SOM inputs are similar in both genotypes (two-way ANOVA, *p* = 0.2627, *F*_(1, 37)_ = 1.293, *n*_Eu-6w_ = 8, *n*_Eu-8w_ = 11, *n*_Ts65Dn-6w_ = 10, *n*_Ts65Dn-6w_ = 12). Eu, euploid; GC, granule cell; GCL, granule cell layer; H, hilus; iML, inner molecular layer; IPSC, inhibitory postsynaptic current; Kyn, kynurenic acid; mML, medial molecular layer; oML, outer molecular layer; SML, stratum lacunosum moleculare; SOM, somatostatin.

A previous study in the PFC indicated that SOM interneuron-mediated inhibition to more distal compartments could also cause enhanced GABAergic transmission in Ts65Dn mice (de Zorilla San Martin et al., 2020). To directly test whether SOM interneuron-mediated dendritic inhibition is altered in the DG of Ts65Dn mice, we optogenetically stimulated these interneurons expressing ChR2. The field of illumination was directed to the outer ML for dendritic inhibition to selectively stimulate synaptic inputs of SOM interneurons innervating the distal dendrites of GCs (Figures 2A–D). Dendritic IPSCs of GCs were measured in the presence of Kyn.

Figures 2E,F represent the input–output relation of dendritic IPSCs from SOM interneurons in response to increasing laser intensity in 6-week-old mice. There was a significant main effect of the laser light intensity on the amplitude (*p* < 0.0001, *F*_(3, 63)_ = 72.30, mixed-effect analysis, n_Eu_ = 11, n_Ts65Dn_ = 12). In addition, there was significant light intensity by genotype interaction (*p* = 0.0012, *F*_(3, 48)_ = 6.175, mixed-effect analysis, n_Eu_ = 8, n_Ts65Dn_ = 10; Figure 2F). However, IPSC amplitudes recorded in 8-week-old were not different between Eu and Ts65Dn mice (laser intensity genotype interaction, *p* = 0.6, *F*_(3, 63)_ = 0.61, mixed-effect analysis, n_Eu_ = 11, n_Ts65Dn_ = 12; Figure 2G; Supplementary Figure S3). Thus, while the IPSC amplitude from SOM inputs were reduced in 6-week-old animals, this phenomenon was no longer present in 8-week-old mice. This result may be explained by a delayed maturation of SOM inputs to distal dendrites of GCs in Ts65Dn mice. However, these results do not support the hypothesis that SOM interneuron inputs to the distal dendritic tree of GCs contribute to increased GABAergic transmission in the DG of Ts65Dn mice.

**Figure 3:**
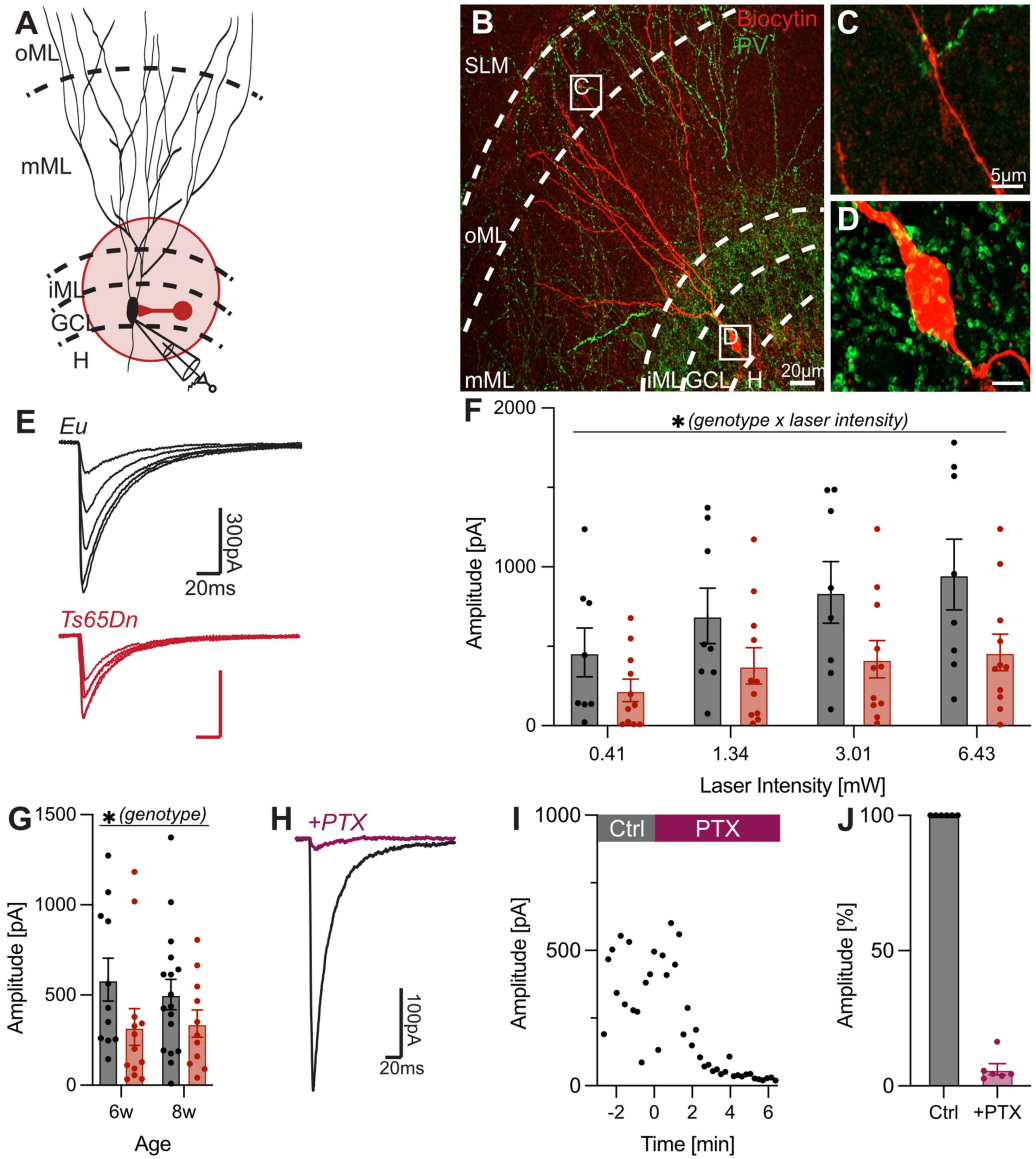
Reduced input of PV interneurons onto GCs in the DG of Ts65Dn mice. **(A)** Experimental design. Optogenetic stimulation in the GCL was used to activate synaptic inputs from PV interneurons indicated by a red schematic with both its soma and axon in the GCL. PV interneurons were optically stimulated in the GCL and IPSCs were recorded from GCs at a holding potential of −80 mV. **(B)** A biocytin-filled GC is pictured in red and PV interneurons which express YFP, are shown in green. The green YFP signal mainly labels the dendrites of PV interneurons in the ML and PV axons and synapses in the GCL. **(C)** The distal dendrite of the GC in the oML is shown in detail. Fibers of PV interneurons do not enclose the GC dendrite. **(D)** In the GCL, the YFP signal labels the dense axon network of PV interneurons including putative synapses that are formed onto the soma of the GC. **(E)** Representative IPSCs evoked by optical stimulation of PV interneurons and recorded from GCs in 8-week-old Eu and Ts65Dn mice. **(F)** Input–output relationship is shown by plotting IPSC amplitude over the strength of stimulation. Mixed-effect analysis (repeated measure) demonstrated a pronounced effect of the genotype × laser intensity interaction (*p* = 0.0097, *F*_(3, 51)_ = 4.221, *n*_Eu_ = 8, *n*_Ts65Dn_ = 11). **(G)** IPSCs measured in 6- and 8-week-old mice in response to the same laser intensity. Two-way ANOVA indicated a significant effect of the genotype (*p* = 0.0340, *F*_(1, 48)_ = 4.763, *n*_Eu-6w_ = 11, *n*_Eu-8w_ = 17, *n*_Ts65Dn-6w_ = 13, *n*_Ts65Dn-8w_ = 11). **(H)** Representative IPSCs before and after application of 100 μM PTX, blocking GABA_A_Rs. IPSCs were evoked at an intensity of 0.35 mW. **(I)** PTX reduces the IPSC amplitude over time in Eu mice. After approximately 5 min the response was completely suppressed. **(J)** Presence of PTX reduced the response up to 94% (Ctrl = 100%; +PTX = 93.8 ± 2.1%, *n* = 6). Ctrl, control; DG, dentate gyrus; Eu, euploid; GC, granule cell; GCL, granule cell layer; H, hilus; iML, inner molecular layer; IPSC, inhibitory postsynaptic current; mML, medial molecular layer; oML, outer molecular layer, PTX, picrotoxin; PV, parvalbumin; SLM, stratum lacunosum moleculare.

The perisomatic compartment receives prominent inhibition that is primarily mediated by PV interneurons (Kraushaar and Jonas, 2000; Hu et al., 2014). PV interneuron-mediated synaptic inputs onto GCs have not been directly investigated in the DG of Ts65Dn mice previously. In mice expressing ChR2 in PV interneurons, we targeted the field of illumination to the GCL with the recorded GC in the center (Figures 3A–D). The addition of 100 μM PTX fully blocked the evoked IPSCs confirming that they were GABAergic (Figures 3H–J).

Inputs were evoked with increasing stimulation intensity in slices from 8-week-old Eu and Ts65Dn littermates (Figures 3E,F). There was a significant effect of the laser light intensity on IPSC amplitude (*p* < 0.0001, *F*_(3, 51)_ = 33.50, mixed-effect analysis, n_Eu_ = 8, n_Ts65Dn_ = 11), which, however, appeared rather weak in Ts65Dn mice. Interestingly, IPSCs evoked by an optogenetic stimulation of PV interneurons were reduced in Ts65Dn mice (Figure 3F). Thus, there was a significant interaction between laser intensity and genotype (*p* = 0.0097, *F*_(3, 51)_ = 4.22, mixed-effect analysis, n_Eu_ = 8, n_Ts65Dn_ = 11). Similar results were obtained in a second independent set of data recorded in 6-week-old animals (Figure 3G; Supplementary Figure S4). Combining both datasets confirmed a significant effect of the genotype (*p* = 0.03, *F*_(1, 48)_ = 4.7, two-way ANOVA, n_Eu-6w_ = 11, n_Eu-8w_ = 17, n_Ts65Dn-6w_ = 13, n_Ts65Dn-6w_ = 11). Hence, these results indicate that PV interneurons do not contribute to increased GABAergic transmission in the DG of Ts65Dn mice and that PV interneuron-mediated perisomatic inhibition may be reduced instead.

### Properties of proximal dendritic GABAergic inputs are clearly distinct from SOM and PV interneuron-mediated inhibition

The results so far suggest that electrically evoked proximal dendritic inputs are enhanced in Ts65Dn mice, while optogenetically evoked inputs from SOM and PV interneurons were either unchanged or reduced. To investigate whether electrically evoked proximal dendritic inputs were indeed distinct from inputs from SOM and PV interneurons, we investigated the kinetics of the inputs and their short-term plasticity. These properties are characteristic for specific sets of synapses and depend on the presynaptic neuron of a synapse, postsynaptic receptor composition and location within the dendritic tree (Savanthrapadian et al., 2014; Schulz et al., 2018).

**Figure 4:**
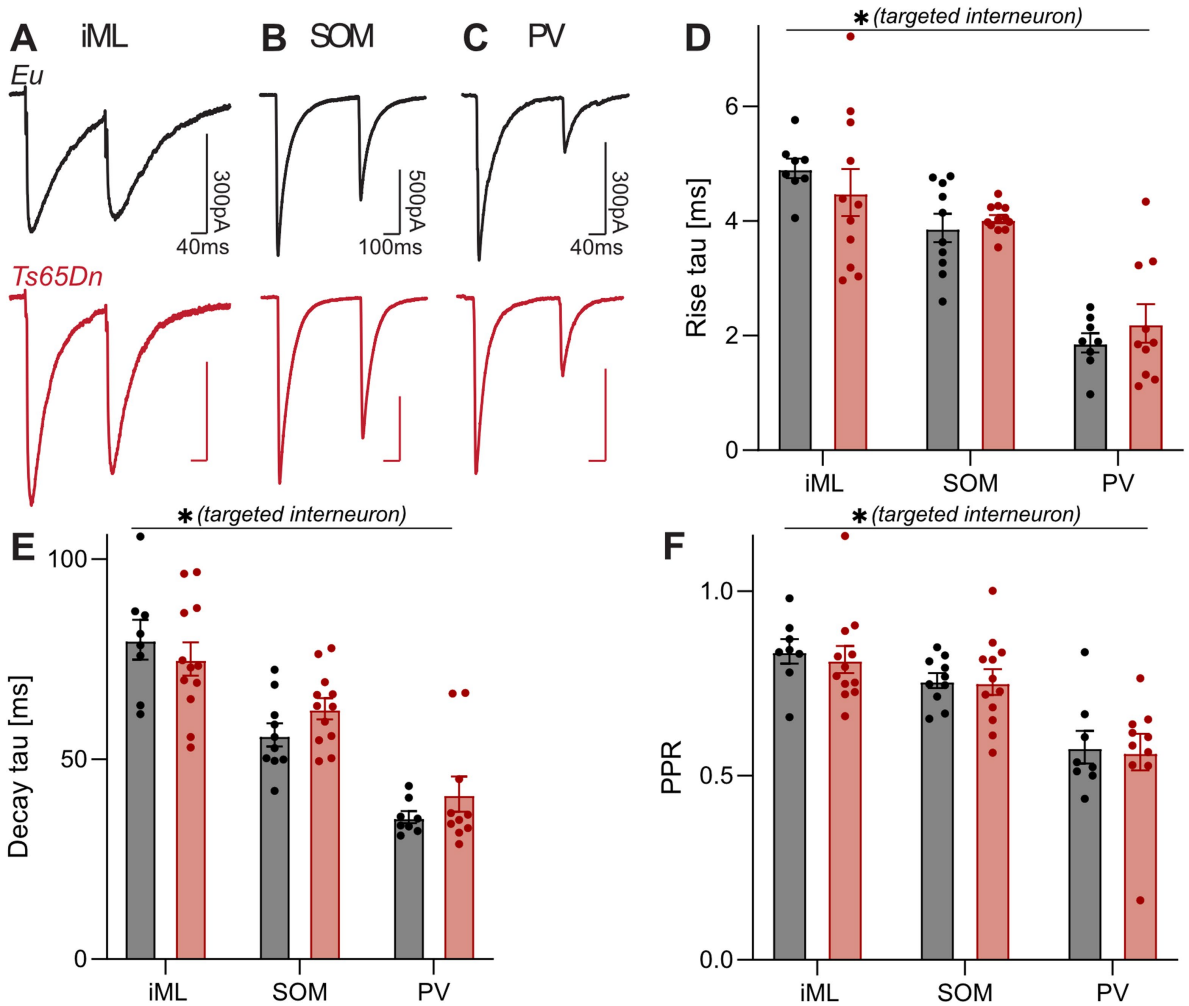
Pronounced difference in the kinetics and short-term plasticity of GABAergic inputs recruited by optogenetic and electrical stimulation. **(A)** Representative IPSCs in response to a double-pulse stimulation evoked by electrical stimulation in the iML, optogenetic stimulation of SOM interneurons in the oML **(B)** and PV interneurons in the GCL **(C)**. **(D)** Group means of the rise time of the first IPSCs for the two genotypes and the three stimulations. Two-way ANOVA reveals a significant main effect of the targeted interneuron population (*p* < 0.0001, *F*_(2, 54)_ = 48.12, iML: *n*_Eu_ = 8, *n*_Ts65Dn_ = 11, SOM: *n*_Eu_ = 11, *n*_Ts65Dn_ = 12, PV: *n*_Eu_ = 8, *n*_Ts65Dn_ = 10), but no main effect of the genotype (*p* = 0.9997, *F*_(1, 54)_ = 1.061e-007, two-way ANOVA). **(E)** Group means of the decay time constant *τ* of the second IPSC. A two-way ANOVA showed a significant effect of the targeted interneuron population (*p* < 0.0001, *F*_(2, 56)_ = 55.09). There was no main effect of genotype (*p* = 0.40, *F*_(1, 56)_ = 0.73, two-way ANOVA). **(F)** Short-term plasticity was assessed by the PPR. A two-way ANOVA of the grouped data showed a significant main effect of targeted interneuron population (*p* < 0.0001, *F*_(2, 56)_ = 22.79). There was no main effect of genotype (*p* = 0.73, *F*_(1, 56)_ = 0.12, two-way ANOVA). iML, inner molecular layer; Eu, euploid; PPR, paired-pulse-ratio; PV, parvalbumin; SOM, somatostatin.

We used two-way ANOVAs to assess genotype-specific differences in synaptic properties. For none of the properties, we observed significant genotype specific effects (Figure 4; Supplementary Table S1). Therefore, we pooled data across genotypes for further comparisons of synaptic properties between different targeted presynaptic interneurons. First, we evaluated the rise time of the optically and electrically evoked IPSCs from 20 to 80% of the peak amplitude to baseline (Figures 4A–D). IPSCs elicited by the three different targeted stimulations showed significantly different rise times (*p* < 0.0001, *F*_(2, 57)_ = 2.64, one-way ANOVA) (Figure 4D). Electrical stimulation in the iML evoked IPSCs with significantly slower rise time (Eu = 4.92 ± 0.17 ms, *n* = 8; Ts65Dn = 4.50 ± 0.41 ms, *n* = 11) than by PV interneurons (Eu = 1.88 ± 0.17 ms, *n* = 8; Ts65Dn = 2.21 ± 0.34 ms, *n* = 10; *p* < 0.0001, Sidak’s multiple-comparisons test). They also tended to be slower than IPSCs mediated by SOM interneurons (Eu = 3.95 ± 0.23 ms, *n* = 11; Ts65Dn = 4.04 ± 0.07 ms, *n* = 12; *p* = 0.03, Sidak’s multiple-comparisons test).

Next, we checked the decay time constant *τ*, which was measured from 95% of the peak amplitude to the baseline. Again, one-way ANOVA demonstrated a significant difference between IPSCs from the three different stimulations (*p* < 0.0001, *F*_(2, 59)_ = 1.66) (Figures 4A–C,E). IPSCs of the iML stimulation showed the slowest τ (Eu = 79.86 ± 4.98 ms, *n* = 8; Ts65Dn = 74.86 ± 3.81 ms, *n* = 13). IPSCs generated by stimulating PV interneurons showed the fastest decay (Eu = 35.49 ± 1.51 ms, *n* = 8; Ts65Dn = 41.23 ± 4.42 ms, *n* = 10). SOM interneurons evoked IPSCs were indicative of a slower time constant τ (Eu = 55.82 ± 2.66 ms, *n* = 11; Ts65Dn = 62.62 ± 2.65 ms, *n* = 12) than PV interneuron- and faster than iML stimulation-evoked ones (PV vs. SOM: *p* < 0.0001; SOM vs. iML: *p* < 0.0001, Sidak’s multiple-comparisons test). Based on the kinetics, we suggest that the type of interneuron contributing to the observed over-inhibition has rather slow output synapses.

The paired-pulse ratio (PPR) was used to examine synaptic short-term plasticity. There were significantly different PPR values for the three stimulations (*p* < 0.0001, *F*_(2, 59)_ = 0.27, one-way ANOVA) (Figures 4A–C,F). The highest PPR was calculated for the iML stimulation (Eu = 0.84 ± 0.03, *n* = 8; Ts65Dn = 0.81 ± 0.03, *n* = 13). A similar ratio was calculated for SOM interneuron-mediated inputs (Eu = 0.75 ± 0.02, *n* = 11; Ts65Dn = 0.75 ± 0.03, *n* = 12; *p* = 0.18, Sidak’s multiple-comparisons test). A paired-pulse depression, with the lowest PPR, was identified for synapses of PV interneurons (Eu = 0.58 ± 0.04, *n* = 8; Ts65Dn = 0.56 ± 0.05, *n* = 10) (Figures 4A,D) (SOM vs. PV: *p* < 0.0001, Sidak’s multiple-comparisons test).

Together, the results from the kinetics and PPR analyses suggested that neither PV nor SOM interneurons contributed significantly to the GABAergic inputs evoked by our electrical stimulation in the iML. We did not notice any genotype-specific effects for the short-term plasticity of the different stimulations. Hence, our results suggest that the presynaptic properties of GABAergic synapses are not the major cause of an altered efficiency of GABAergic transmission in the DG of 8-week-old Ts65Dn mice. Electrical properties with slower rise time and weaker PPD than PV inputs have been reported for CCK interneurons (Hefft and Jonas, 2005). Based on the position of our electrical stimulation as well as the observed pronounced difference of kinetics and PPRs, we hypothesized that inhibition mediated by CCK interneurons may be increased in Ts65Dn mice.

For a more direct assessment of the involvement of CCK interneurons, we tested the effect of CB1 receptor activation on the IPSCs evoked in the iML. CB1 receptors are known to be strongly and selectively expressed on the presynaptic terminals of CCK interneurons where they contribute to regulation of presynaptic release properties (Katona et al., 1999). However, bath application of 1 µM ACEA did not induce a clear reduction of IPSCs evoked by electrical stimulation in iML, although there was a non-significant trend to a reduction in recordings from Eu mice (Supplementary Figure S5). While these results could not support our hypothesis that increased inhibition in the DG was mediated by CCK interneurons, it may be possible that they reflect differences in the ventral versus the dorsal hippocampus (see Discussion). We proceeded to evaluate the source of increased synaptic inhibition in the iML of Ts65Dn mice by immunohistochemical methods.

### Increased number of CB1^+^ and constant PV^+^ terminals in the DG of Ts65Dn mice

**Figure 5:**
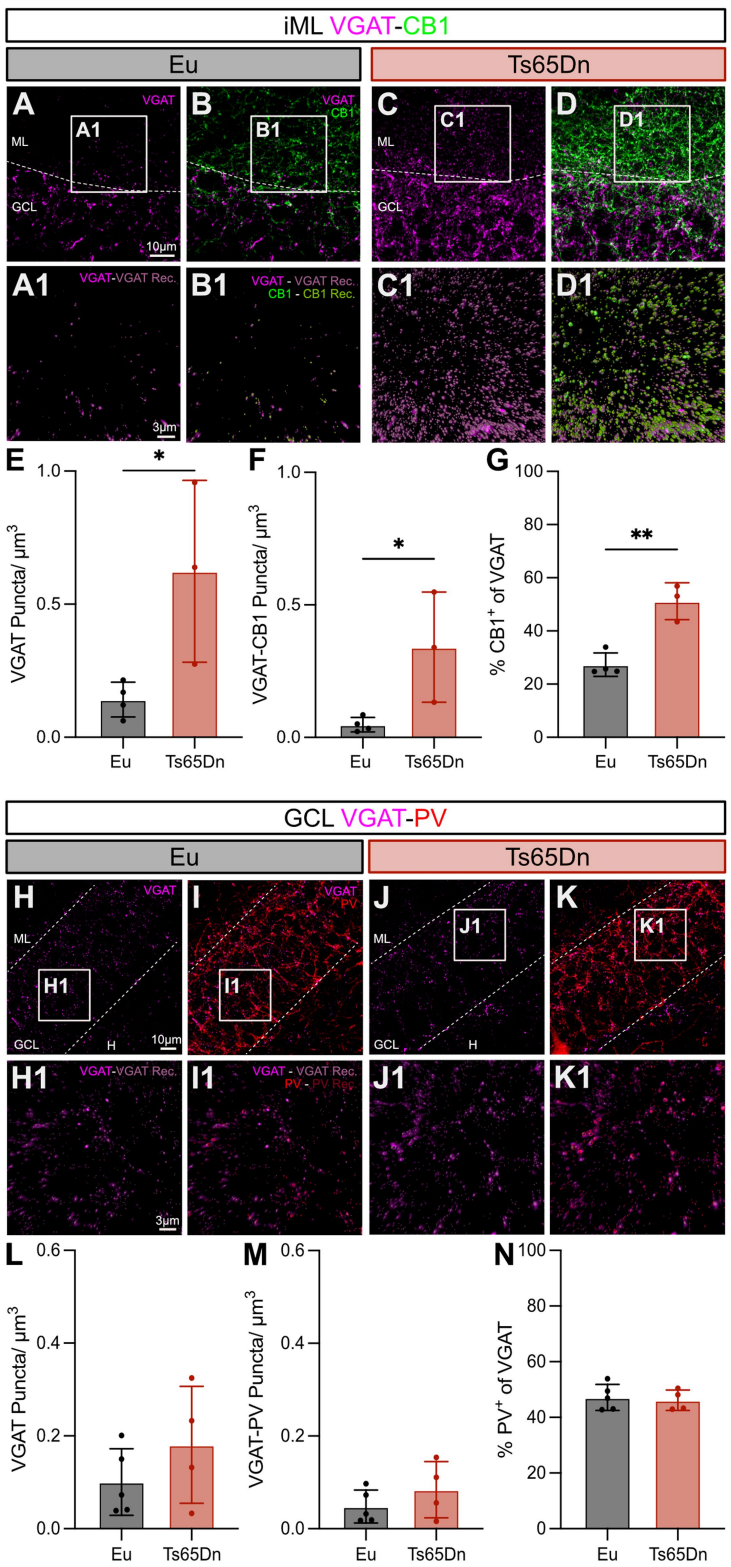
Strong increase of CB1^+^ inhibitory synapses in the iML, while the density of PV^+^ terminals is not altered in the GCL of Ts65Dn mice. **(A–D)** Representative sections within the DG immunostained for VGAT **(A,C)** and CB1 **(B,D)** in Eu **(A,B)** and Ts65Dn mice **(C,D)**. VGAT is highly expressed in the GCL and to a lower extent in the adjacent ML. CB1 signal is mainly found in the iML, where it overlaps with inhibitory synapses stained with VGAT. **(A1–D1)** Detailed images within the iML (insets marked in **A–D**). 3D reconstruction of VGAT **(A1,C1)** and VGAT-CB1 **(B1,D1)**, using the spot analysis tool in Imaris, reflects reliable results in both genotypes. **(E)** Quantification of inhibitory synapses shown as puncta per μm^3^. Means per animal were analyzed and revealed a significantly enhanced density of VGAT puncta in Ts65Dn mice (*p* = 0.04, *F*_(2, 3)_ = 27.09, *n*_Eu_ = 4, *n*_Ts65Dn_ = 3, unpaired *t*-test two-tailed). **(F)** Group means per animal of the density of VGAT-CB1 double positive reconstructed puncta per μm^3^. Increase in VGAT-CB1 puncta in the iML reached significance in Ts65Dn mice (*p* = 0.03, *F*_(2, 3)_ = XXX, unpaired *t*-test two-tailed). **(G)** Relative number of CB1 puncta from all inhibitory VGAT puncta. Massive increase, with about 20% more inhibitory CB1^+^ synapses in the iML of Ts65Dn mice (*p* = 0.003, *F*_(2, 3)_ = 2.43, unpaired *t*-test two-tailed). **(H–K)** Representative images of DG with their focus on the GCL. VGAT is highly expressed in this layer **(H,J)** and overlaps with PV labeling. Basket-like shapes can be identified with the PV staining **(I,K)** in Eu **(H,I)** and Ts65Dn littermates **(J,K)**. **(H1–K1)** Higher magnification used for visualization of VGAT **(H1,J1)** and VGAT-PV synapses **(I1,K1)** in the two genotypes. Insets are marked in **H–K**. 3D reconstruction was performed with the spot analysis tool of Imaris and led to solid results. **(L)** Quantification of VGAT synapses within the GCL did not differ between genotypes (*p* = 0.27, *F*_(3, 4)_ = 3.09, *n*_Eu_ = 5, *n*_Ts65Dn_ = 4, unpaired *t*-test two-tailed). **(M)** Analysis of VGAT-PV double positive puncta per μm^3^ did as well show no alteration in Ts65Dn mice compared to Eu littermates (*p* = 0.29, *F*_(3, 4)_ = 2.93, unpaired *t*-test two-tailed). **(N)** Relatively the number of PV^+^ synapses of all VGAT^+^ synapses was for both genotypes at a similar level (*p* = 0.73, *F*_(4, 3)_ = 1.61, unpaired *t*-test two-tailed). CB1, cannabinoid receptor type 1; DG, dentate gyrus; Eu, euploid; GCL, granule cell layer; H, hilus; iML, inner molecular layer; ML, molecular layer; PV, parvalbumin; VGAT, vesicular GABA transporter.

An increased number of inhibitory synapses in the iML of Ts65Dn mice has been reported by Martínez-Cué et al. (2013). CCK interneurons densely innervate the iML and highly express the endocannabinoid receptor 1 (CB1) in their axons (McDonald, 2021; Morozov and Freund, 2003). To clarify if CCK interneurons might contribute to enhanced inhibitory synapse density in the iML, we used double-labeling of CB1 with the inhibitory synaptic marker VGAT (Figures 5A–D). This allowed a specific reconstruction of CCK^+^ synapses in the DG (Figures 5A1–D1). CB1 expression was restricted to the iML and outer GCL for both genotypes. 3D-reconstruction revealed that the number of VGAT^+^ inhibitory synapses (Eu = 0.14 ± 0.03 puncta/μm^3^, *n* = 4; Ts65Dn = 0.62 ± 0.20, *n* = 3, *p* = 0.04, *F*_(2, 3)_ = 27.09, unpaired t-test two-tailed) (Figure 5E) and CCK interneuron synapses (CB1^+^ and VGAT^+^) were strongly increased in Ts65Dn mice (Eu = 0.048 ± 0.014 puncta/μm^3^, *n* = 4; Ts65Dn = 0.34 ± 0.12, *n* = 3, *p* = 0.03, *F*_(2, 3)_ = 58.24, unpaired t-test two-tailed) (Figure 5F). Furthermore, the relative proportion of CB1^+^ puncta of VGAT-labeled presynaptic terminals was significantly increased in Ts65Dn mice (Eu = 27.31 ± 2.22%, *n* = 4; Ts65Dn = 51.17 ± 3.99%, *n* = 3, *p* = 0.003, *F*_(2, 3)_ = 2.43, unpaired t-test two-tailed) (Figure 5G). These results suggest that the increased inhibition in the iML of Ts65Dn mice may be caused by an increased number of CCK^+^ synapses.

In a second step we investigated the terminals of PV interneurons with double labeling for PV and VGAT (Figures 5H–K). The PV signal was concentrated in the GCL and we observed the characteristic basket-like patterns of PV terminals targeting the soma of GCs (Figures 5I,K). Reconstruction of VGAT^+^ synapses (Eu = 0.10 ± 0.03, *n* = 5; Ts65Dn = 0.18 ± 0.06, *n* = 4, *p* = 0.27, *F*_(3, 4)_ = 3.09, unpaired t-test two-tailed) (Figures 5H1,J1,L) and specifically PV^+^ inhibitory synapses (PV^+^ and VGAT^+^) revealed that their densities were not altered in the GCL of Ts65Dn mice (Eu = 0.048 ± 0.016, *n* = 5; Ts65Dn = 0.084 ± 0.030, *n* = 4, *p* = 0.29, *F*_(3, 4)_ = 2.93, unpaired t-test two-tailed) (Figures 5I1,K1,M). The relative number of PV synapses was consistent between the two genotypes (Eu = 47.21 ± 2.09%, *n* = 5; Ts65Dn = 46.18 ± 1.84%, *n* = 4, *p* = 0.73, *F*_(4, 3)_ = 1.61, unpaired t-test two-tailed) (Figure 5N). Together, these results support a specific increase of CCK^+^ synapses on GCs in Ts65Dn mice.

## Discussion

This study provides new insights into how PV, SOM and CCK interneurons may contribute to increased GABAergic signaling in the DG of Ts65Dn mice. Cell-specific optogenetic stimulation showed that PV and SOM interneurons do not contribute to increased inhibition in the DG of Ts65Dn mice. In contrast, CCK interneurons which densely innervate the iML and form CB1^+^ synapses on the proximal dendrites of GCs may contribute to increased inhibition in Ts65Dn mice, as supported by a larger amplitude of IPSCs evoked by electrical stimulation in the iML and increased CB1^+^ positive presynaptic puncta in the iML.

To the best of our knowledge, no study to date has investigated the specific contribution of CCK interneurons to altered inhibition in Ts65Dn mice. Di Franco et al. (2022) were the first to observe a higher number of CB1 positive putative interneurons in the hilus of Ts65Dn mice. CCK interneurons are known to strongly innervate the iML of the DG (Hefft and Jonas, 2005). Within the iML, CB1 is highly expressed in the terminals of CCK interneurons and only to a small fraction in astrocytes (Freund et al., 2003; Navarrete and Araque, 2008). In agreement with previous studies (Martínez-Cué et al., 2013), we counted more VGAT-positive presynaptic GABAergic terminals in the iML of Ts65Dn mice. Furthermore, we could demonstrate that the density of inhibitory CB1^+^ synapses in the iML was increased by about 20% in Ts65Dn mice compared to Eu littermates. This indicates that there are more synapses from CCK interneurons onto the proximal dendrites of GCs. This conclusion is also supported by increased GABAergic synaptic inputs evoked in the iML, which showed no indication of altered synaptic release properties or kinetics. As direct optogenetic stimulation of other non-CCK interneurons did not show increased synaptic inputs, our results indicate that there is an increased density of GABAergic synapses specifically from CCK interneurons in the iML that significantly contributes to altered inhibition in the DG of Ts65Dn mice.

One important limitation of the current study is that we could not directly confirm that increased inhibition in the iML of Ts65Dn mice is mediated by CCK interneurons. A direct pharmacological test of the involvement of CB1 receptor-responsive presynaptic terminals from CCK interneurons remained inconclusive (Supplementary Figure S5). This may indicate that other interneuron subtypes than CCK interneurons strongly contribute to increased GABAergic inhibition in the iML of Ts65Dn mice. This would be in agreement with overall increased VGat puncta in the iML of Ts65Dn mice that was not restricted to CB1+ VGat puncta (Figure 5). In addition, recent research highlights that effects of endocannabinoid signaling significantly differ in the ventral compared to the dorsal hippocampus (Serra et al., 2024; Tsotsokou et al., 2025). Among other differences, the ventral hippocampus is subject to different dopamine signaling (Kempadoo et al., 2016; Nordin et al., 2025; Dubovyk and Manahan-Vaughan, 2019). Dopamine signaling has been shown to modulate endocannabinoid signaling such that CB1-mediated depression of inhibition is switched to enhancement by higher dopamine D2 signaling in the globus pallidus (Caballero-Florán et al., 2016). Therefore, it is conceivable that CB1-mediated action on presynaptic release may also have different consequences in the ventral compared to the dorsal hippocampus. This important question will have to be addressed in future experiments.

Earlier work suggested that an increased number of GABAergic interneurons in the cortex and hippocampus of Ts65Dn mice may contribute to altered inhibition in Ts65Dn mice (Chakrabarti et al., 2010; Pérez-Cremades et al., 2010; Hernández-González et al., 2015). More recent studies showed that specifically dendritic inhibition was increased in the CA1 and the PFC of Ts65Dn mice (Schulz et al., 2019; de Zorilla San Martin et al., 2020). In the PFC, the SOM^+^ Martinotti cells were identified as an important source to an increased inhibition in Ts65Dn mice (de Zorilla San Martin et al., 2020), whereas in CA1 the type of interneuron was not defined (Schulz et al., 2019). In the current study, we used optogenetic stimulation to investigate the contribution of SOM and PV interneurons to altered inhibition in the DG of Ts65Dn mice. However, optogenetically evoked inputs from SOM interneurons were decreased specifically at 6 weeks of age. This indicates a maturation dependent delay in distal dendritic inhibition which may be related to delayed dendritic maturation of GCs in the DG of Ts65Dn mice (Uguagliati et al., 2021). The observation of clearly decreased optogenetically evoked inhibition from PV interneurons is more puzzling. In contrast to decreased optogenetically evoked IPSCs from PV interneurons, the density of PV synapses in Ts65Dn did not differ from Eu littermates. As the PPRs remained unchanged, alterations of presynaptic release probability were unlikely to cause differences in the evoked IPSCs between genotypes. Altered axonal excitability of PV interneurons in the DG of Ts65Dn mice could be one factor that may explain lower recruitment of PV axon to light stimulation while total PV synapse numbers were not changed. The specific reasons for the discrepancy between our electrophysiological observation on PV interneuron inputs onto GCs and analyses of PV synapse density remain unclear at present. Irrespective of the underlying causes for partially decreased optogentically evoked inhibition from SOM and PV interneurons, these results clearly demonstrate that inhibition by these major interneuron subtypes does not contribute to increased inhibition in the DG of Ts65Dn mice.

The DG as part of the hippocampus plays an important role for memory formation and is responsible for pattern separation (Leutgeb et al., 2007; McHugh et al., 2007; Myers and Scharfman, 2011; Madar et al., 2019). Pattern separation is required to distinguish between similar experiences, which are encoded by incoming signals from the entorhinal cortex into the DG as multimodal patterns. The GCs are suggested to disperse these multimodal patterns by sparsely firing and thereby minimizing the similarity between experiences (Leutgeb et al., 2007; Madar et al., 2019). The different interneuron subpopulations within the DG tightly control its activity and contribute to the sparse firing of GCs (Hu et al., 2014; Elgueta and Bartos, 2019). Preclinical data indicates that memory deficits in DS are linked to an enhanced GABA signaling in the DG (Fernandez et al., 2007; Kleschevnikov et al., 2004; Kleschevnikov et al., 2012; Martínez-Cué et al., 2013) and may particularly affect spatial context-dependent learning (Hyde et al., 2001; Smith et al., 2014). In agreement with these observations in mouse models, particular difficulty in recalling spatial locations when contextual information was salient has been reported in people with DS (Clark et al., 2017). Recently, altered expression of endocannabinoids, and CB1 receptor, together with increased number of CB1-positive neurons in the hilus have been linked to the disease pathophysiology in Ts65Dn mice (Di Franco et al., 2022; Navarro-Romero et al., 2019). In addition, results from pharmacogenetic inhibition of ventral hippocampal CCK interneurons indicate that these neurons may act as regulators of spatial contextual reward learning (Nguyen et al., 2024). Based on our present results, we propose that enhanced GABAergic signaling mediated by CCK interneurons in the DG of Ts65Dn mice may lead to an altered activation of GCs and impaired long-term potentiation that encodes for spatial contextual information. Hence, the specificity of the output of the DG along the trisynaptic loop could be decreased and interfere with memory formation. This is expected to impair hippocampus functions like spatial coding and place preference learning in DS (Witton et al., 2015).

In light of these new results, correcting the altered GABAergic microcircuitry in the DG appears more challenging than previously thought. While direct modulation of the GABAergic system may be too unspecific, targeting the endocannabinoid system has been proposed previously (Navarro-Romero et al., 2019). CB1 receptor knockout in GABAergic neurons leads to cognitive deficits similar to those observed in Ts65Dn mice (Albayram et al., 2016) making CB1 receptor a potentially interesting target. Therefore, finding a targeted approach that may alleviate the most prominent GABAergic circuit deficits underlying learning impairments in DS remains a promising and important goal.

In conclusion, the present study highlights the important role of CCK interneurons on the inhibitory-excitatory imbalance in DS. We identified these interneurons as the main contributors to an increased GABAergic synapse density in the iML of the DG in a mouse model of DS that may be responsible for impaired learning and memory.

## Data Availability

The original contributions presented in the study are included in the article/Supplementary material, further inquiries can be directed to the corresponding author.
