# Supplementary material for: Increased dendritic inhibition of dentate gyrus granule cells in a mouse model of Down syndrome
Source: Front Cell Neurosci. 2026 Feb 26;20:1714586. doi: 10.3389/fncel.2026.1714586 (PMC12981062; doi:10.3389/fncel.2026.1714586)
Supplement: Supplementary file 1 [file Presentation_1.pdf]

SUPPLEMENTARY DATA

Increased dendritic inhibition of dentate gyrus granule cells in a mouse model of Down syndrome

Nicole Gutmann[1,2,3], Ute Häussler[1,4], Anabel Mersi[1], Marie Follo[5], Josef Bischofberger[3], Jan M. Schulz[3,6*]

1 Translational Epilepsy Research, Department of Neurosurgery, Medical Center - University of Freiburg, Faculty of Medicine, Freiburg, Germany

2 Faculty of Biology, University of Freiburg, Freiburg

3 Department of Biomedicine, University of Basel, Basel, Switzerland

4 BrainLinks-BrainTools, University of Freiburg, Freiburg, Germany

5 Department of Medicine I, Lighthouse, Core Facility, Medical Center - University of Freiburg, Faculty of Medicine, University of Freiburg, Freiburg, Germany

6 Roche Pharma Research & Early Development, Neuroscience & Rare Diseases Discovery, Roche Innovation Center Basel, F. Hoffmann-La Roche Ltd, Basel, Switzerland

*correspondence to j.schulz@unibas.ch

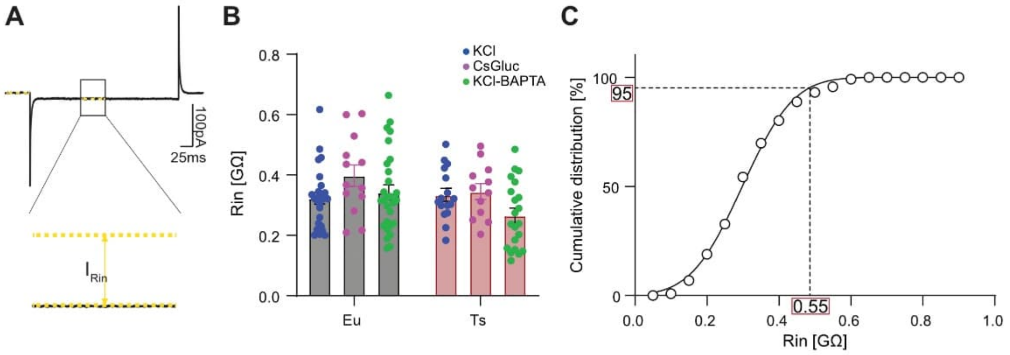

Figure S1: Analysis of the input resistance in 8-week-old mice. A) Representative recording of a GC after break-in in response to a -5 mV pulse. Zoom-in showing how the current step for the calculation was measured. B) Group means of Eu and Ts65Dn mice for the three different intracellular solutions that were used in the study. The genotype did not affect the input resistance (R_{in}) measurements. C) Data Pooled from Eu and Ts65Dn of all experiments. Cumulative distribution over the input resistance. The plot indicates that 95% of all values were lower or equal to 0.55 GΩ. To obtain a homogeneous data set for the evaluation of genotype-specific differences of synaptic GABAergic inputs, only GCs with input resistances of ≤ 550 MΩ were included in the study. All other GCs were considered to be too immature.

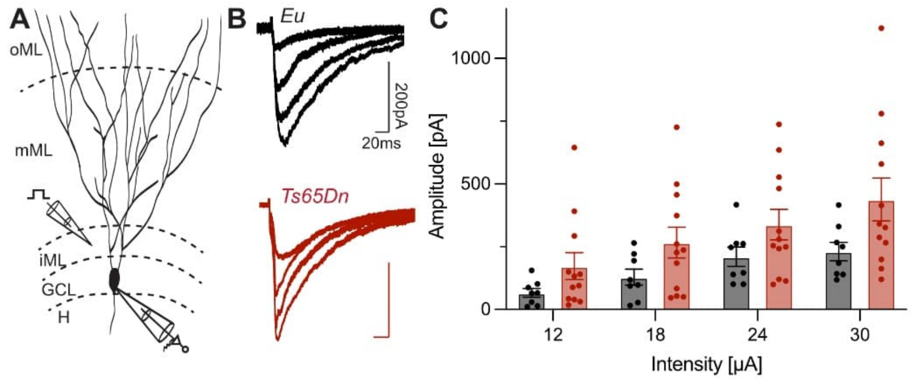

Figure S2: IPSCs evoked by electrical stimulation in the iML tend to be larger in 8-week-old Ts65Dn mice. A) Schematic illustration of the stimulation site within the iML and recording from GCs. Holding potential was set to -80 mV, all recordings were done with the KCl-BAPTA intracellular solution and in presence of 3 mM Kyn. B) Representative traces in response to the electrical iML stimulation measured in 8-week-old Eu and Ts65Dn mice. C) Input-output relation indicated by plotting amplitude over stimulated current. For all stimulation intensities the response seems to be stronger in Ts65Dn mice. Mixed-effect analysis reveals a nearly significant effect of the genotype ($p = 0.0820$, $F_{(1, 18)} = 3.393$, $n_{Eu} = 8$, $n_{Ts65Dn} = 12$). Eu euploid; GC granule cell; GCL granule cell layer; H hilus; iML inner molecular layer; IPSC inhibitory postsynaptic current; Kyn kynurenic acid; mML medial molecular layer; oML outer molecular layer.

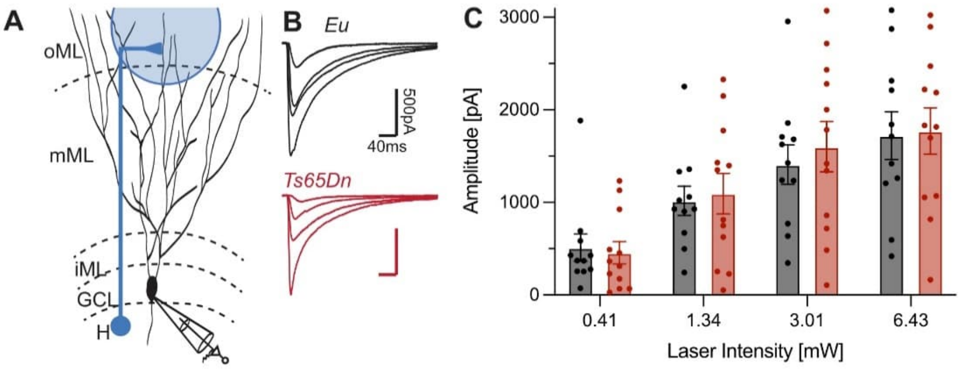

Figure S3: Inputs of SOM interneurons onto GCs in fully mature 8-week-old are not different between Ts65Dn and Eu mice. A) Schematic illustrating the experimental design, with optogenetic stimulation of SOM inputs onto the distal dendrites of the recorded GC and soma location of SOM interneuron in the hilus. Holding potential was set to -80 mV and experiments were performed in presence of 3 mM Kyn. F) Representative IPSCs in response to the optical stimulation of SOM synapses in the oML in 8-week-old Eu and Ts65Dn mice. C) Grouped data of the input-output relation. For all stimulation intensity the amplitude of optogenetically evoked IPSCs was indistinguishable between Eu and Ts65Dn mice ($p = 0.8132$, $F_{(1, 21)} = 0.05724$, $n_{Eu} = 11$, $n_{Ts65Dn} = 12$, mixed-effect analysis). Eu euploid; GC granule cell; GCL granule cell layer; H hilus; iML inner molecular layer; IPSC inhibitory postsynaptic current; Kyn kynurenic acid; mML medial molecular layer; oML outer molecular layer; SOM somatostatin.

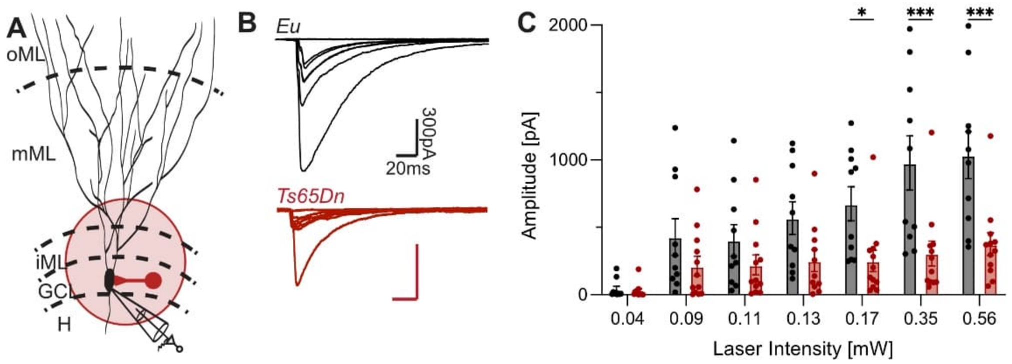

Figure S4: Somatic inhibition mediated by PV interneurons is reduced in 6-week-old Ts65Dn mice. A) Illustration of the experimental design. The field of illumination was set to target the GCL with the recorded GC in its center. Red schematic indicates the location of the PV interneuron. GCs were held at a potential of -80 mV. B) Representative responses evoked by optogenetic stimulation of PV interneuron terminals in the GCL in 6-week-old Eu and Ts65Dn mice. C) IPSC amplitude is plotted over the laser intensity. Mixed-effect analysis reveals a reduction of PV inputs in Ts65Dn mice ($p = 0.0093$, $F_{(1, 20)} = 8.284$, $n_{Eu} = 10$, $n_{Ts65Dn} = 12$). Eu euploid; GC granule cell; GCL granule cell layer; H hilus; iML inner molecular layer; IPSC inhibitory postsynaptic current; mML medial molecular layer; oML outer molecular layer; PV Parvalbumin.

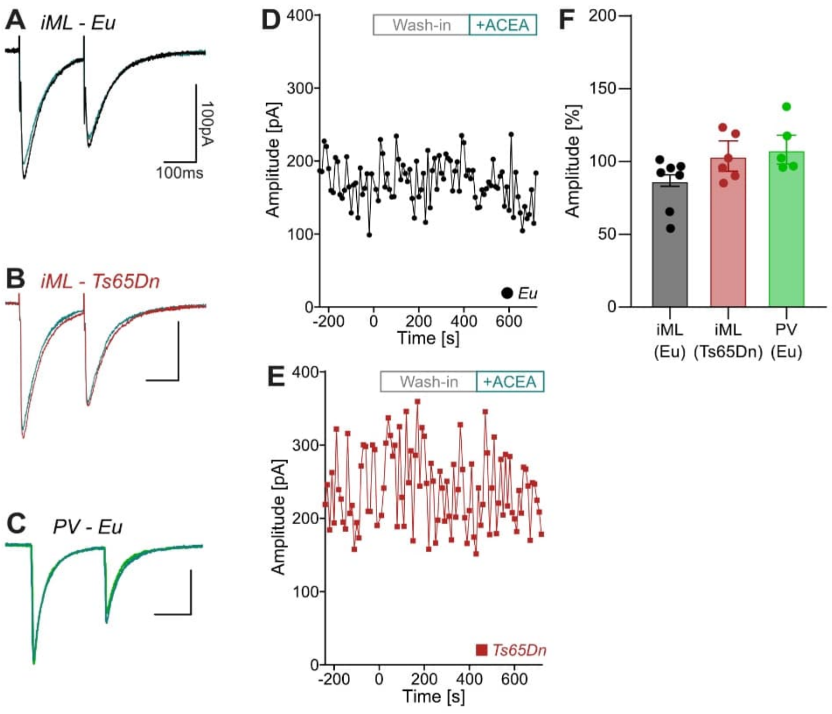

Figure S5: Bath application of the CB1 receptor agonist ACEA and its effect on the evoked IPSCs. Representative mean IPSCs evoked by electrical stimulation in the iML in Eu (black, A) and Ts65Dn mice (red, B) and optogenetic stimulation of PV inputs (green, C). The response averaged over 5 min in the presence of 1 µM ACEA is indicated in blue. D-E) Amplitudes of individual IPSCs evoked in iML plotted over time for a representative recording from an Eu (D) and a Ts65Dn mouse (E). Wash-in of ACEA started at 0. The time interval for the measurement of the full drug effect of ACEA is indicated. F) iML-evoked IPSC amplitude in presence of ACEA tended to be decreased in Eu animals ($p = 0.064$, one sample t-test, two-tailed), whereas IPSCs were not affected in Ts65Dn mice or for PV inputs in Eu mice (see Table S1).

Table S1. Results of all statistical analyses.

Figure	Description	Statistical test	Number of samples	Effects	P value	F (DFn, DFd)	Sidak's multiple comparison
**Figure 1 - iML**	Input-output relation 6-week-old mice	mixed-effect analysis repeated measure	n(Eu) = 10, n(Ts65Dn) = 9	Intensity	<0.0001	F (2.008, 34.14) = 57.01	Eu vs. Ts: 10 - p = 0.1405; 15 - p = 0.3567; 20 - p = 0.1139; 25 - p = 0.2599; 30 - p = 0.0782
				Genotype	0.0176	F (1, 17) = 6.917	
				Intensity x Genotype	0.0502	F (4, 68) = 2.504	
	Comparison 6-week and 8-week old mice	2way ANOVA non-repeated measure	6 weeks: n(Eu) = 11, n(Ts65Dn) = 9<br>8 weeks: n(Eu) = 8, n(Ts65Dn) = 12	Interaction	0.7778	F (1, 35) = 0.08084	Eu vs. Ts: 6w - p = 0.0381; 8w - p = 0.0904
				Age	0.029	F (1, 35) = 5.182	
				Genotype	0.0029	F (1, 35) = 10.23	
	Gabazine Application	2way ANOVA non-repeated measure	n(Eu) = 4, n(Ts65Dn) = 4	Interaction	0.503	F (1, 12) = 0.4768	Eu: p<0.0001; Ts: p<0.0001
				Genotype	0.503	F (1, 12) = 0.4768	
				Drug	<0.0001	F (1, 12) = 751.3	
**Figure 2 - SOM**	Input-output relation 6-week-old mice	mixed-effect analysis repeated measure	n(Eu) = 8, n(Ts65Dn) = 10	Intensity	<0.0001	F (1.335, 21.36) = 82.61	Eu vs. Ts: 0.41 - p>0.9999; 1.34 - p>0.9999; 3.01 - p = 0.353; 6.43 - p = 0.1545
				Genotype	0.1729	F (1, 16) = 2.036	
				Intensity x Genotype	0.0012	F (3, 48) = 6.175	
	Comparison 6-week and 8-week old mice	2way ANOVA non-repeated measure	6 weeks: n(Eu) = 8, n(Ts65Dn) = 10<br>8 weeks: n(Eu) = 11, n(Ts65Dn) = 12	Interaction	0.1854	F (1, 37) = 1.821	Eu vs. Ts: 6w - p = 0.201; 8w - p = 0.9839
				Age	0.1613	F (1, 37) = 2.043	
				Genotype	0.2627	F (1, 37) = 1.293	
**Figure 3 - PV**	Input-output relation 8-week-old mice	mixed-effect analysis repeated measure	n(Eu) = 8, n(Ts65Dn) = 11	Intensity	<0.0001	F (3, 51) = 33.50	Eu vs. Ts: 0.41 - p = 0.672; 1.34 - p = 0.4137; 3.01 - p = 0.1548; 6.43 - p = 0.0702
				Genotype	0.0803	F (1, 17) = 3.460	
				Intensity x Genotype	0.0097	F (3, 51) = 4.221	
	Comparison 6-week and 8-week old mice	2way ANOVA non-repeated measure	6 weeks: n(Eu) = 11, n(Ts65Dn) = 13<br>8 weeks: n(Eu) = 17, n(Ts65Dn) = 11	Interaction	0.6027	F (1, 48) = 0.2745	Eu vs. Ts: 6w - p = 0.1329; 8w - p = 0.4118
				Age	0.7481	F (1, 48) = 0.1043	
				Genotype	0.034	F (1, 48) = 4.763	
	Picrotoxin application	unpaired t-test two-tailed	n = 6	Drug	<0.0001	Infinity, 5, 5	no post-hoc
**Figure 4 - Kinetics + PPR**	Rise time	2way ANOVA non-repeated measure	iML: n(Eu) = 8, n(Ts65Dn) = 11	Interaction	0.3869	F (2, 54) = 0.9664	iML vs. SOM: Eu - p = 0.0488; Ts - p = 0.4812<br>iML vs. PV: Eu- p<0.0001; Ts - p<0.0001<br>SOM vs. PV: Eu - p<0.0001; Ts - p<0.0001
			SOM: n(Eu) = 11, n(Ts65Dn) = 12	Stimulation	<0.0001	F (2, 54) = 48.57	
			PV: n(Eu) = 8, n(Ts65Dn) = 10	Genotype	0.9997	F (1, 54) = 1.061e-007	

	Decay	2way ANOVA non-repeated measure	iML: n(Eu) = 8, n(Ts65Dn) = 13	Interaction	0.1980	F (2, 56) = 1.667	iML vs. SOM: Eu - p<0.0001; Ts - p = 0.0280<br>iML vs. PV: Eu- p<0.0001; Ts - p<0.0001<br>SOM vs. PV: Eu - p = 0.0009; Ts - p = 0.0002
			SOM: n(Eu) = 11, n(Ts65Dn) = 12	Stimulation	<0.0001	F (2, 56) = 55.09	
			PV: n(Eu) = 8, n(Ts65Dn) = 10	Genotype	0.3962	F (1, 56) = 0.7309	
	Paired-pulse-ratio	2way ANOVA non-repeated measure	iML: n(Eu) = 8, n(Ts65Dn) = 13	Interaction	0.9120	F (2, 56) = 0.09225	iML vs. SOM: Eu - p = 0.2938; Ts - p = 0.5267<br>iML vs. PV: Eu - p = 0.0001; Ts - p< 0.0001<br>SOM vs. PV: Eu - p = 0.0087; Ts - p = 0.0013
			SOM: n(Eu) = 11, n(Ts65Dn) = 12	Stimulation	<0.0001	F (2, 56) = 22.79	
			PV: n(Eu) = 8, n(Ts65Dn) = 10	Genotype	0.7301	F (1, 56) = 0.1202	
	Pooled data of Eu and Ts65Dn for rise	one-way ANOVA	iML n = 19, SOM n = 23; PV = 18	Stimulation	<0.0001	F (2, 57) = 2.640	iML vs. SOM: p = 0.0330; iML vs. PV: p<0.0001; SOM vs. PV: p<0.0001
	Pooled data of Eu and Ts65Dn for decay	one-way ANOVA	iML n = 21, SOM n = 23; PV = 18	Stimulation	<0.0001	F (2, 59) = 1.663	iML vs. SOM: p<0.0001; iML vs. PV: p<0.0001; SOM vs. PV: p<0.0001
	Pooled data of Eu and Ts65Dn for PPR	one-way ANOVA	iML n = 21, SOM n = 23; PV = 18	Stimulation	<0.0001	F (2, 59) = 0.2778	iML vs. SOM: p = 0.1329; iML vs. PV: p<0.0001; SOM vs. PV: p<0.0001
**Figure 5 - Reconstruction**	VGAT puncta iML	unpaired t-test two-tailed	n(Eu) = 4, n(Ts65Dn) = 3	Density	0.0360	F (2, 3) = 27.09	no post-hoc
	VGAT-CB1 puncta iML	unpaired t-test two-tailed	n(Eu) = 4, n(Ts65Dn) = 3	Density	0.0347	F (2, 3) = 58.24	no post-hoc
	% CB1 of VGAT	unpaired t-test two-tailed	n(Eu) = 4, n(Ts65Dn) = 3	%	0.0025	F (2, 3) = 2.432	no post-hoc
	VGAT puncta GCL	unpaired t-test two-tailed	n(Eu) = 5, n(Ts65Dn) = 4	Density	0.2670	F (3, 4) = 3.086	no post-hoc
	VGAT-PV puncta GCL	unpaired t-test two-tailed	n(Eu) = 5, n(Ts65Dn) = 4	Density	0.2940	F(3, 4) = 2.930	no post-hoc
	%PV of VGAT	unpaired t-test two-tailed	n(Eu) = 5, n(Ts65Dn) = 4	%	0.7307	F(4, 3) = 1.610	no post-hoc
**Figure S1 - Rin**	Rin - Comparison of intracellular solutions	2way ANOVA	KCl: n(Eu) = 26; n(Ts65Dn) = 16	Interaction	0.1855	F (2, 110) = 1.711	KCl vs. CsGluc: Eu- p = 0.1471; Ts- p = 0.9922<br>KCl vs. KCl-BAPTA: Eu - p = 0.9000; Ts - p = 0.1885<br>CsGluc vs. KCl-BAPTA: Eu - p = 0.3636; Ts - p = 0.1493
			CsGluc: n(Eu) = 13; n(Ts65Dn) = 12	Genotype	0.0742	F (1, 110) = 3.250	
			KCl-BAPTA: n(Eu)= 28; n(Ts65Dn) = 21	Intracellular solution	0.0538	F (2, 110) = 3.001	
**Figure S2 - iML**	Input-output relation - 8-week-old mice	mixed-effect analysis repeated measure	n(Eu) = 8, n(Ts65Dn) = 12	Intensity	<0.0001	F (3, 54) = 21.20	Eu vs. Ts: 12 - p = 0.6337; 18 - p = 0.3824; 24- p = 0.4555; 30 - p = 0.0729
				Genotype	0.0820	F (1, 18) = 3.393	
				Intensity x Genotype	0.3353	F (3, 54) = 1.155	
**Figure S3 - SOM**	Input-output relation - 8-week-old mice	mixed-effect analysis repeated measure	n(Eu) = 8, n(Ts65Dn) = 12	Intensity	<0.0001	F (3, 63) = 72.30	Eu vs. Ts: 0.41- p = 0.9996; 1.34 - p = 0.9983; 3.01- p = 0.9474; 6.43 - p = 0.9997
				Genotype	0.8132	F (1, 21) = 0.05724	

				Intensity x Genotype	0.6102	F (3, 63) = 0.6113	
**Figure S4 - PV**	Input-output relation - 6-week-old mice	mixed-effect analysis repeated measure	n(Eu) = 10, n(Ts65Dn) = 12	Intensity	<0.0001	F (6, 118) = 23.01	Eu vs. Ts: 0.04 - p>0.9999; 0.09 - p = 0.6882; 0.11 - p = 0.8377; 0.13 - p = 0.2100; 0.17 - p = 0.0384; 0.35 - p = 0.0001; 0.56 - p = 0.0002
				Genotype	0.0093	F (1, 20) = 8.284	
				Intensity x Genotype	<0.0001	F (6, 118) = 6.996	
**Figure S5 - ACEA**	Effect ACEA	one sample t-test	n(iML-Eu) = 7, n(iML-Ts655Dn) = 6, n(PV-Eu) = 5	iML Eu	0.0643	t=2.263, df=6	
				iML Ts65Dn	0.7463	t=0.3420, df=5	
				PV-Eu	0.2789	t=1.252, df=4	
